# Design principles underpinning the regulatory diversity of protein kinases

**DOI:** 10.1098/rstb.2012.0015

**Published:** 2012-09-19

**Authors:** Krishnadev Oruganty, Natarajan Kannan

**Affiliations:** 1 Department of Biochemistry and Molecular Biology, University of Georgia, Athens, GA, USA; 2 Institute of Bioinformatics, University of Georgia, Athens, GA, USA

**Keywords:** phylogeny, allostery, genomics, bioinformatics, signalling, regulation

## Abstract

Protein phosphorylation in eukaryotes is carried out by a large and diverse family of protein kinases, which display remarkable diversity and complexity in their modes of regulation. The complex modes of regulation have evolved as a consequence of natural selection operating on protein kinase sequences for billions of years. Here we describe how quantitative comparisons of protein kinase sequences from diverse organisms, in particular prokaryotes, have contributed to our understanding of the structural organization and evolution of allosteric regulation in the protein kinase domain. An emerging view from these studies is that regulatory diversity and complexity in the protein kinase domain evolved in a ‘modular’ fashion through elaboration of an ancient core component, which existed before the emergence of eukaryotes. The core component provided the conformational flexibility required for ATP binding and phosphoryl transfer in prokaryotic kinases, but evolved into a highly regulatable domain in eukaryotes through the addition of exaggerated structural features that facilitated tight allosteric control. Family and group-specific features are built upon the core component in eukaryotes to provide additional layers of control. We propose that ‘modularity’ and ‘conformational flexibility’ are key evolvable traits of the protein kinase domain that contributed to its extensive regulatory diversity and complexity.

## 1. Introduction

Eukaryotic protein kinases (EPKs) catalyse the transfer of the terminal phosphate group from ATP (γ-phosphate) to the hydroxyl group of a serine, threonine or tyrosine residue in protein substrates. The catalytic core that performs this process is highly conserved and remarkably specific to substrates in signalling pathways. Since signalling pathways control important cellular processes such as transcription, cell cycle progression, differentiation and apoptosis (see reviews [1–4]), precise regulation of protein kinase activity is critical for the survival of the eukaryotic cell. Indeed, crystal structures of several EPKs solved in both active and inactive forms reveal the conformational flexibility of the catalytic core (reviewed in [5–11]) and its role in regulating protein kinase activity. For example, cyclin-dependent kinases (Cdk2), which participate in cell cycle progression, are subject to multiple layers of control as they switch from an inactive ‘off’ state to an active ‘on’ state. During cell cycle progression, newly formed CDK–cyclin complexes initially accumulate in an inactive state in which Cdk2 is phosphorylated on two adjacent residues (T14 and Y15) in the nucleotide-binding P-loop. Dephosphorylation of these two residues and phosphorylation of a threonine (T160) in the activation loop [12] leads to full activation of CDK–cyclin complexes and cell cycle progression. Each of these regulatory events invokes specific conformational changes within the catalytic core [7,13,14], which also occur in the activation process of various other EPKs [5,15].

Over the past few years, sequence-based search procedures, crystal structures and biochemical studies have revealed several protein kinase-like (PKL) families in bacteria, archaea and in lower eukaryotes that are distantly related to the EPKs. Some of these families include aminoglycoside phosphotransferases in pathogenic bacteria [16], lipopolysaccharide kinases in Gram-negative bacteria [17] and choline kinases [18], collectively called CAK kinases [19]. The catalytic cores of these families are strikingly similar in structure to the catalytic core of eukaryotic protein kinases [18,20], despite very low sequence similarity, and they can together be grouped as EPK-like kinases (ELKs) [21]. Although ELKs have not been as extensively studied as EPKs, existing literature on some of the ELK families such as aminoglycoside kinase (APH) indicate that, unlike EPKs, APH can phosphorylate both protein substrates [22] and aminoglycosides [23]. Similarly, the function of APH in pathogenic bacteria is to confer resistance to naturally occurring fungal antibiotics [24], which strikingly differs from the function of EPKs in signalling pathways. Furthermore, crystal structures of APH solved in nucleotide bound and unbound forms do not display the dramatic conformational changes typically observed in EPKs [23].

Both EPKs and ELKs are more distantly related to several distinct classes of atypical kinases (APKs) [25] that specifically phosphorylate certain protein and small molecule substrates. Examples of APKs include the eukaryotic elongation factor 2 kinase [26], phosphoinositide 3-kinase (PI3 kinase) [27] and the isocitrate dehydrogenase kinase (ICDH kinase; also called AceK) [28]. The substrate specificity of AceK has recently been studied using crystal structures [29], and it has been shown that AceK recognizes the entire ICDH dimer rather than short peptide regions from ICDH. Such specificity is achieved by protrusion of AceK substrate recognition helix into the active site of ICDH dimer [28]. Another example of APK substrate specificity comes from structures solved for actin–fragmin kinase [26], which suggests evolution of an elaborate complementary surface that specifically binds to the actin–fragmin dimer. This specific binding of substrates by APKs is in contrast to EPKs and ELKs, which generally display substrate diversity [30,31].

Given the evolutionary relationship between EPKs, ELKs and APKs and the striking differences in substrate specificity and regulatory aspects of these three classes of kinases, one can ask the following important questions. First, what sequence and structural features are typical of the catalytic core of EPKs and ELKs, but not of APKs, and how do these features facilitate precise regulation of EPKs in signalling pathways? Second, what features are common to EPKs and ELKs and how do they relate to their common functions? Third, can we specifically pinpoint these features given the amount of sequence data [19,32] now available on all three groups of kinases?

Here, we describe how quantitative comparisons of the evolutionary constraints acting on EPK, ELK and APK sequences and structures have provided insights into the modular organization and evolution of regulation and substrate specificity in the protein kinase domain. We define the core structural features shared by EPKs, ELKs and APKs, and show that EPKs and ELKs have diverged from APKs through the addition of structural features that contribute to the conformational flexibility of the catalytic core. The EPK–ELK shared features are further elaborated in EPKs through the addition of flexible loops, such as the activation loop, which provides a framework for allosteric regulation by phosphorylation. We show that group and family-specific motifs within EPKs are built upon flexible regulatory segments, such as the activation loop, to provide additional layers of regulation.

## 2. What are the minimum structural requirements for adopting the protein kinase-like fold?

**Figure 1.**
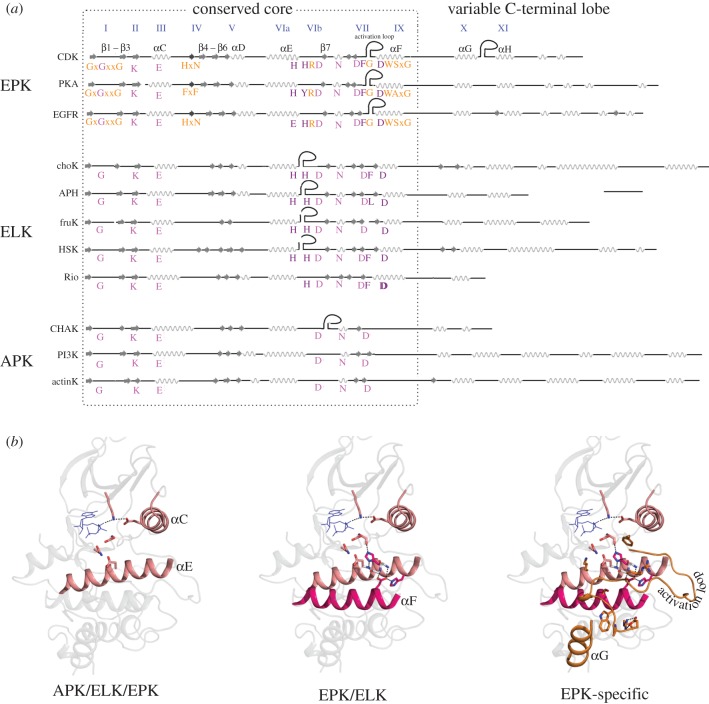
Conservation of secondary structures and residues in the protein kinase superfamily. A schematic of kinases belonging to the protein kinase superfamily is shown (*a*). The secondary structures are proportional to the length found in the crystal structure, but the loop lengths are not proportional. The insert segments are marked with a stem loop-type structure. Secondary structures within inserts are not shown. The conserved residues are shown below the secondary structure diagram for each protein. The sub-domains to which each conserved pattern belong are given above the secondary structure in blue-coloured roman numbers. The colouring scheme for the alignment is as follows: residues conserved in all three classes of kinases are shown in light pink, residues conserved only in ELKs and EPKs are shown in magenta and EPK-specific residues are shown in orange. The C-lobe secondary structures that are not conserved are given on the right and are not aligned. The structures used for generating the alignment are: CDK (1QMZ) [35], PKA (1ATP) [36], EGFR (2GS2) [37], choK or choline kinase (2IG7), APH or aminoglycoside kinase (3R78), fruK or fructosamine kinase (3F7W), HSK or homoserine kinase (1FWK) [38], Rio kinase (1ZP9) [39], CHAK or TRP channel kinase (1IA9) [40], PI3K or phosphoinositide kinase (3T8M) and actinK or actin–fragmin kinase (1CJA) [26]. (*b*) The location of the conserved residues and secondary structures at the three levels of conservation. The residues are shown in sticks representation and the colouring scheme followed is all oxygen atoms are coloured red and all nitrogen atoms are coloured blue. The carbon atoms of conserved residues and secondary structures conserved in all three classes (APK, ELK and EPK) are coloured light pink. The carbon atoms of conserved residues and secondary structures conserved in ELKs and EPKs but not in APKs are coloured magenta. The carbon atoms of conserved residues and secondary structures conserved only in EPKs, but not in other classes, are coloured orange. The structure figures were generated in PyMOL based on the crystal structure of cyclin-dependent kinase (pdb: 1QMZ).

EPKs adopt the same fold as ELKs and APKs despite sharing very low sequence similarity [25,33]. This raises the question as to what the minimum requirements are for adopting the PKL fold. Comparison of representative EPKs, ELKs and APKs indicate that among the 12 hallmark motifs (or sub-domains) of the EPK domain (as defined by Hanks & Hunter [34]), only a few of the motifs/residues are commonly shared by EPKs, ELKs and APKs [33]. These motifs, shown in figure 1, correspond to: (i) a glycine within the ATP-binding G-loop (sub-domain I), (ii) a lysine/arginine in beta sheet 3 (sub-domain II) that binds ATP, (iii) glutamate in C-helix (sub-domain III) that coordinates with the beta sheet 3 lysine/arginine, (iv) aspartate in the catalytic loop (sub-domain VIb) that serves as a catalytic base, (v) a magnesium ion coordinating asparagine in the catalytic loop (sub-domain VIb) [36], and (vi) a magnesium coordinating aspartate in the beginning of the activation segment (sub-domain VII) [41,42]. These residues/motifs, which mostly occur in the N-terminal ATP-binding lobe (figure 1), appear to define the minimum structural requirements for adopting the PKL fold [25,33]. It should, however, be noted that the N-terminal ATP-binding lobe of PKLs is also known to share structural similarity with ATP-grasp fold enzymes [40]. Thus, there may be a subset of these essential residues that are sufficient and necessary to form the conserved core of the PKL fold. Additional comparisons of kinases with ATP-grasp fold enzymes will therefore be necessary to fully define the core structural features of the PKL fold.

## 3. Is the c-terminal substrate-binding lobe an independent folding unit?

**Figure 2.**
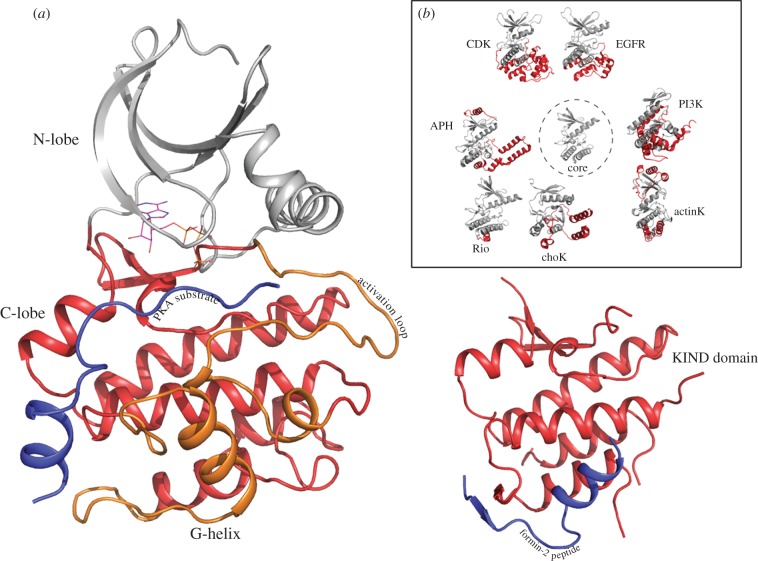
The kinase C-lobe as a distinct module. (*a*) PKA structure and KIND domain structure are compared to show the similarity of the PKA C-lobe to the independently existing KIND domain (pdb: 2YLE) [43]. The substrate-binding G-helix is absent in the KIND domain, but a peptide from the KIND interacting protein, formin2, binds to the region analogous to the G-helix of kinase C-lobe. (*b*) The inset shows the variation in C-lobe structure in multiple kinases. The kinases shown here correspond to a subset of the kinases shown in figure 1.

Unlike the N-terminal ATP-binding lobe, the C-terminal lobe (helices G-H-I in Cdk2) is highly variable across EPKs, ELKs and APKs (figure 1). The C-terminal lobe adopts distinct three-dimensional structures in the three classes, reflecting on the substrate differences (figure 2*a,b*). The C-lobe was also suggested to be structurally related to a distinct domain named ‘kinase non-catalytic C-lobe domain’ (KIND) based on remote homology searches [44]. Specifically, the KIND domain was reported to exist independently in the actin nucleation factor Spir, Ras guanine exchange factor [45], protein tyrosine phosphatase basophil-like (PTP-BL/BAS) and the multi-PDZ domain protein FRMPD2 [44]. Recently, the crystal structure of the KIND domain from Spir actin nucleators was determined, and was found to resemble the C-terminal lobe of the kinase domain as predicted by sequence analysis [43,46]. As shown in figure 2*a*,*b*, the overall organization of the helices (E,F,H,I) in the kinase C-lobe resembles the organization of helices found in the Spir KIND domain, except the G-helix, which is different in the Spir KIND domain and the C-lobe of kinases (figure 2*a*). Notably, the peptide corresponding to the KIND interaction protein, formin, adopts a conformation analogous to the G-helix of the kinase C-lobe in the crystal structure of the KIND–formin peptide complex (figure 2*a*). This suggests that the C-lobe of the kinase is a distinct functional unit, which can carry out protein interaction functions independent of the N-terminal ATP-binding lobe. Such separation of functions (ATP binding in N-lobe and substrate binding in C-lobe) would lend the kinase domain a substantial degree of flexibility/robustness in evolving multiple substrate specificities within the same catalytic framework (figure 2*b*).

## 4. The epk–elk structural component provides a flexible framework for coupling atp and substrate-binding sites

**Figure 3.**
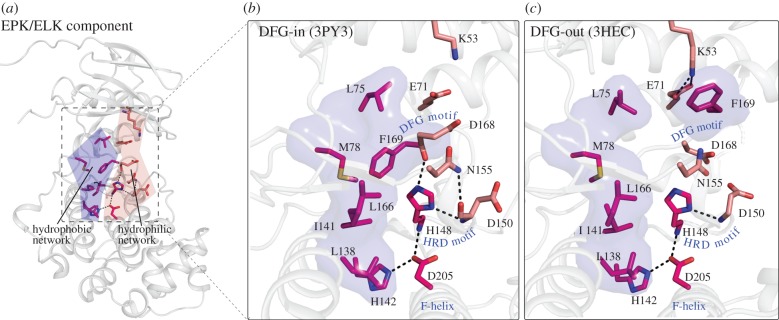
EPK–ELK component residues shown in the context of active and inactive p38alpha crystal structures. The EPK/ELK component is composed of two networks (shown in (*a*)). The hydrophobic interactions are shown as surface blobs and the residues participating in interactions are shown in sticks representation. (*b*) The residues interacting in the two networks in p38alpha (pdb: 3PY3) [47] in an active conformation. (*c*) The same residues are also shown in another p38alpha structure when it is in an inactive conformation (3HEC) [48]. The reversible breaking of both the networks is a hallmark of EPK–ELK component residues.

EPKs and ELKs share certain sequence and structural features in common that are not present in APKs (figure 1). One such feature is the F-helix in the C-lobe of the kinase domain [33]. In addition to the F-helix, two networks of interacting residues also distinguish EPKs and ELKs from APKs. One is the hydrophobic network comprising L75, M78, L138, I141, L166, F169 residues (figure 3), and the other is a network of polar interactions formed by H142, H148 and D205 (figure 3). The F-helix and the hydrophobic and hydrogen bond networks together constitute the EPK–ELK structural component that most distinguishes EPKs and ELKs from APKs [33]. Below, we review recent structural, computational and functional studies that provide insights into the EPK–ELK structural component.

### (a) Hydrophobic network

The hydrophobic network in the EPK–ELK structural component is formed by a contiguous network of closely packed interactions that couple the N-terminal ATP-binding lobe and the C-terminal substrate-binding lobe (figure 3). The hydrophobic network is structurally conserved across diverse EPK and ELK structures and some of the residues in the network were also identified using the local spatial pattern (LSP) method by comparing representative EPK crystal structures [49]. Based on the LSP method, Kornev *et al.* [49,50] defined some of the hydrophobic network residues as the ‘regulatory spine’ because they observed that the hydrophobic network is assembled in active kinases, but disassembled in the inactive forms (figure 3). Consistent with the regulatory role for the hydrophobic network, mutation of the spine residue resulted in kinase inactivation in some tyrosine kinases [51,52]. The conservation of the hydrophobic network in ELKs suggests that it performs a similar regulatory role; however, this hypothesis needs to be tested through structural and biochemical studies.

### (b) Hydrogen bonding network

The hydrogen bonding network in the EPK–ELK structural component couples the catalytically important DFG and HRD motifs with the F and H helices in the C-lobe (figure 3). However, despite the remarkable conservation of the hydrogen bonding network across diverse EPK and ELK structures, the precise role of this network in EPK–ELK functions is not fully understood. Crystal structure analysis of active and inactive EPKs indicates that the hydrogen bonding network is disrupted in some of the inactive structures, where the catalytically important DFG motif switches from a ‘DFG-in’ conformation to a ‘DFG-out’ conformation (figure 3). The DFG-flip has been suggested to play a role in the catalytic cycle [33,53], and was also shown to alter drug binding at the nucleotide-binding site [54]. Furthermore, NMR studies on p38 MAP kinase indicated that the DFG motif residues are highly mobile in solution [55]. The malleability of the hydrogen bonding network in the ‘DFG-out’ conformations suggests that conformational changes associated with drug–nucleotide binding in the active site can be coupled to the substrate-binding site by the EPK–ELK network. Why would such coupling be important for EPK and ELK functions, but not APK functions? Since ELKs and EPKs phosphorylate multiple substrates, it is likely that the EPK–ELK network evolved to prevent spurious phosphorylation of substrates by providing a flexible communication pathway between the ATP and substrate-binding site. Such a regulatory role also explains the selective conservation of the F-helix in EPKs and ELKs, as the F-helix provides a structural interface between the catalytic and substrate-binding sites, and serves as a scaffold for assembling multiple regulatory signals in EPKs [50,56]. Again, this hypothesis needs to be tested through mutational analysis of EPK–ELK component residues.

### (c) Variations in the EPK–ELK structural component

The EPK–ELK component residues are nearly invariant in both ELKs and EPKs; however, in some EPK families, the EPK–ELK component residues are modified without any apparent change in the catalytic domain structure or fold. For example, in PIM kinases, the F-helix aspartate (D205^p38^ in figure 3) is substituted by alanine. This variation does not alter the folding or structure of PIM kinases [57]. Similarly, in multiple AGC kinases, the HRD motif histidine (H148^p38^ in figure 3) is substituted by tyrosine. Yet another variation is seen in the case of tyrosine kinases, which substitute the E-helix histidine (H142^p38^ in figure 3) with various other polar residues, without any apparent change in the structure. Although the functional relevance of such family-specific variation is currently unclear, it is possible that families that diverge from the canonical EPK–ELK features have evolved alternative mechanisms for coupling between the substrate and ATP-binding sites. Characterizing such family-specific variations will shed further light on the EPK–ELK structural component.

## 5. Epk-specific features built upon the epk–elk structural component

**Figure 4.**
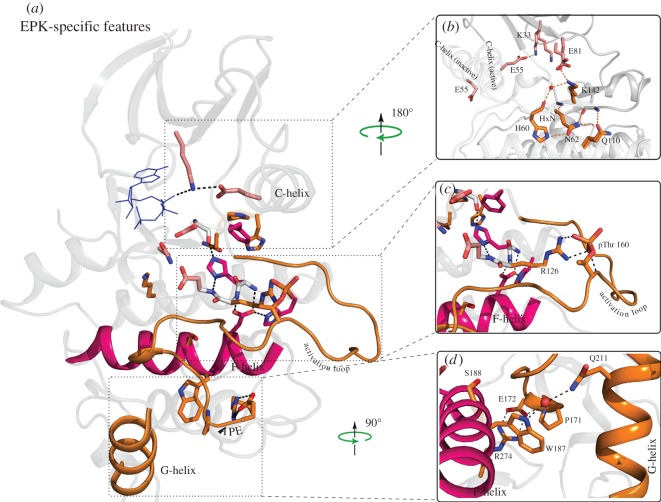
Overview of EPK-specific features. (*a*) Overview of the EPK-specific features and their location with respect to the EPK–ELK component residues and the APK/ELK/EPK-conserved residues. The colouring scheme followed is the same as in figure 1 and this figure was generated based on a CDK structure (1QMZ). EPK-specific features are given in orange colour. (*b*) The C-helix in both active and inactive states in shown. The HxN motif in a beta-turn region is specific to EPKs and participates in regulating the C-helix movement. The colouring scheme for the residues is the same as given in figure 1. (*c*) Activation loop is an EPK-specific feature and a Ser/Thr residue in this loop needs to be phosphorylated for activation of many kinases. An arginine next to the EPK–ELK component histidine (coloured magenta) and catalytic aspartate (coloured light pink) anchors the phospho-threonine in almost all EPKs. (*d*) The G-helix is an EPK-specific feature that binds substrate. The EPK-specific F-helix tryptophan (W187) couples the G-helix and the F-helix via water-mediated hydrogen bonds. Also shown is the interaction between EPK-specific glutamate in APE motif with a conserved arginine (R274) in I helix that couples the activation loop (via APE motif) to the I helix.

EPKs have evolutionarily diverged from ELKs to operate in highly regulated signalling pathways. Indeed, several sequence and structural features distinguish EPKs from ELKs [33], including three exaggerated and/or unusual structural features (figure 4): (i) a solvent-exposed β turn within the loop connecting the C-helix and β4-strand (the αC-β4 loop); (ii) a long activation segment between the β8 strand and F-helix; (iii) a sizeable C-terminal insertion (G-H-I helix) that is involved in substrate binding. These exaggerated structural features are built upon, and tethered to, the EPK–ELK shared component by residues and motifs that are distinctive of EPKs (figure 4). For example, an EPK-specific HxN motif in the αC-β4 loop tethers the flexible C-helix to the EPK–ELK structural component by mediating lobe-bridging hydrogen bond interactions (figure 4*b*). Similarity, an EPK-conserved arginine within the HRD motif tethers the activation loop to the EPK–ELK structural component by coordinating with a phosphorylatable residue in the activation loop (figure 4*c*). Likewise, the EPK-specific W-[SA]-x-G motif in the F-helix tethers the activation loop and substrate-binding G-H-I helices through water-mediated and CH-π interactions (figure 4*d*). Why would such tethering be important for EPK functions? One possibility is that tethering provides an additional layer of regulation beyond the EPK–ELK structural component, and a framework for allosterically coupling distal regulatory sites to the active site. Consistent with this view, mutation of residues in the αC-β4 loop increase fibroblast growth factor receptor 2 (FGFR2) activity by altering C-helix and inter-lobe movement [58]. Likewise, mutation of the HRD-arginine [59], or mutations of residues that tether the activation segment to the F-helix, reduce catalytic activity in protein kinase A (PKA) [60]. It is also worth noting that EPK residues that tether the activation segment to the F-helix (figure 4*d*) are also frequently mutated in congenital disorders [61]. Taken together, these observations indicate that the EPK-specific features play important regulatory roles and are built upon the EPK–ELK structural component to provide additional layers of allosteric control.

## 6. Sequence features contributing to the functional divergence of major epk groups

Although all EPKs share a conserved catalytic and allosteric mechanism, it is also true that each kinase has evolved its own set of regulators and modulators of activity. Such regulators often reside within the same polypeptide as in the case of Src kinase with the SH2 domain regulating its activity [62], or the regulator could reside in another polypeptide such as in the case of cAMP-dependent protein kinase, which is regulated by the R subunit [63]. Clearly, if the regulatory interaction is conserved in evolution, it must leave a mark on the sequence of the kinase involved. The concepts laid out in delineating the features common to all EPKs can also be used for delineating residues that most distinguish one family of kinase from another. Several such attempts have been made in the past and, in this review, the analysis on CMGC and AGC kinases is presented. Both CMGC and AGC kinases consist of members with highly divergent functional roles in the eukaryotic cell. Given the specialized functional niche of these kinases, multiple mechanisms have evolved in just these two classes to regulate their function. Below we describe how identification of sequence motifs unique to the CMGC and AGC groups of kinases has provided insights into the unique modes of regulation in these kinases.

### (a) CMGC kinase-specific residues contribute to substrate specificity and unique modes of allosteric regulation

**Figure 5.**
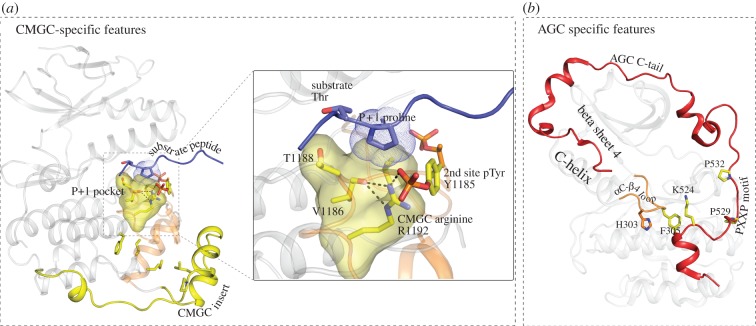
Group-specific features and their interactions with the residues conserved at a higher level. Residues conserved at the group or sub-family levels are coloured yellow. (*a*) CMGC-specific residues contribute to the unique P+1 proline substrate requirement. The P+1 pocket (shown as yellow surface) is formed by hydrophobic residues (indicated in the inset) and the CMGC-arginine. The substrate is shown in deep blue and the P+1 proline is shown in dotted outline. Other CMGC-specific features such as the CMGC insert and residues are shown in yellow. The CMGC-arginine interaction with the phosphotyrosine is shown in the inset. The structure was generated by modelling the CDK substrate (2CCI) [70] onto the substrate-binding pocket of ERK2 (2ERK) [71]. (*b*) AGC-specific features shown in PKCiota structure (pdb: 3A8X). The C-terminal tail (shown in red colour and cartoon representation) is the most distinguishing feature of AGC kinases. Shown in this view is the EPK-specific αC-β4 loop (αC-β4 loop, shown in orange), and the HxF motif specific to the AGC group kinases (HxN in other EPKs). Also shown in sticks representation is a conserved lysine in the C-tail that makes CH-π interactions with the phenylalanine of the HxF motif.

Cdk2, MAPK, GSK3, CLK and related kinase families, collectively called CMGC kinases [32,64], form a closely related group of kinases that have evolutionarily diverged from other EPKs to preferentially phosphorylate substrates with proline at the P+1 position [65]. CMGC kinases are also regulated by a unique regulatory mechanism that involves a phosphorylated tyrosine in the activation loop [66], or a pre-phosphorylated residue in the substrate [67]. They are also known to interact with scaffold proteins via a unique insert segment, called the CMGC insert, located in the C-lobe [68]. Statistical comparisons of the evolutionary constraints imposed on CMGC kinase sequences revealed several residues/motifs that contribute to CMGC kinase functional specialization [69]. The most distinguishing CMGC residue is an arginine (R1192^Erk2^), which confers substrate specificity by stabilizing the P+1 pocket for proline binding (figure 5*a*) [72]. The CMGC-arginine also contributes to regulation by coordinating with a phosphorylatable tyrosine (Y1185) in the activation loop [71]. It is also predicted to coordinate with the pre-phosphorylated phosphate in the SRPK substrate [69]. Likewise, other CMGC kinase residues were proposed to contribute to the unique modes of CMGC kinase regulation by coupling the P+1 pocket to the CMGC kinase-specific insert, which plays a regulatory role in JNK2 [73] and p38 [74]. It is interesting that CMGC kinase-specific features are built upon the EPK-specific activation segment and the substrate-binding G-H-I loop. Such arrangement, presumably, ensures that CMGC kinase-specific scaffolding functions (via the CMGC-insert) are coupled to the substrate-binding functions of the catalytic core (via the activation segment).

### (b) AGC kinase-specific residues contribute to *cis* regulation by the C-terminal tail

PKA, PKB, PKC and related kinases (collectively called AGC kinases) present yet another example of how group-specific motifs are built upon the EPK-specific features to provide additional layers of regulation. Analysis of the evolutionary constraints that most distinguish AGC kinases from other EPKs revealed that the C-terminal tail, which interacts with the catalytic core in PKA, is the most distinguishing feature of AGC kinases [75]. Furthermore, the residues that tether the C-tail to the kinase core were also found to be selectively conserved in AGC kinases [75]. In particular, the EPK-specific HxN motif in the αC-β4 loop is conserved as the HxF motif in AGC kinases (figure 5*b*). This AGC-specific variation allows tethering of the C-tail to the αC-β4 loop by facilitating a CH-π interaction between a C-terminal tail arginine/lysine and the HxF motif phenylalanine (figure 5*b*). Likewise, other AGC kinase-specific motifs tether the C-tail to the N-lobe and active site of the kinase core through interactions that are specifically conserved in AGC kinases [75]. Why would such unique modes of tethering be important for AGC kinase functions? One possibility is that such tethering provides a framework for coupling regulatory functions of the C-tail with catalytic functions of the kinase core. Consistent with this view, deletion of the C-terminal tail [76] or mutation of conserved C-tail motifs alters Hsp90 binding and catalytic activity in PKC [77]. In the Greatwall kinase, similarly, mutations that disrupt the tethering interactions between the C-tail and the kinase core also significantly reduce catalytic activity [78]. The C-tail also contributes to interactions with PDK1, an AGC kinase that phosphorylates other members of the AGC group [79,80].

From the analysis of both AGC kinases and CMGC kinases, it emerges that the kinase scaffold is quite plastic in tolerating multiple regulatory mechanisms. Moreover, each kinase seems to have evolved a unique mode of regulation by conserving motifs that bind different regulatory domains. In both the cases studied, the additional layer of regulation is mediated by insert segments or flanking sequences (CMCG insert near G-helix and the C-tail in AGC kinases). Regulation by such flanking segments has also been shown for tyrosine kinases [81–83], and more specifically for epidermal growth factor receptor (EGFR) kinases, where the juxtamembrane region N-terminal to kinase core plays an activating role [84] and the C-terminal tail plays an auto-inhibitory role [85]. Recent analysis of the evolutionary constraints imposed on the EGFR family of kinases points to the C-terminal tail playing a regulatory role analogous to that of AGC kinases [86]. Thus, addition of regulatory regions as inserts or flanking residues seems to be a common theme in the evolution of functional diversity in EPKs. Further analysis of such inserts and flanking segments and how they have co-evolved with the catalytic core is likely to provide additional functional clues.

## 7. Modularity and design features of the catalytic domain

Modularity is a concept that is widely used to explain the complexity of biological systems. Indeed, the modular recombination of the catalytic domain with diverse regulatory domains has been shown to contribute to functional diversity (reviewed in [87,88]) and evolution of complexity in signalling pathways (reviewed in [89–92]). However, whether or not the catalytic domain itself evolved in a modular fashion has not been previously explored. Quantitative comparisons of primary sequence motifs and three-dimensional structures of EPKs, ELKs and APKs indicate that evolution of the catalytic domain is also modular in that they have evolved through the addition of co-conserved sequence motifs that contribute to the catalytic domain diversity and complexity. However, unlike protein modules, which are known to fold and function as independent units, it is unclear as to whether co-conserved sequence motifs can function as independent units, as suggested in other signalling domains [93–96]. Nevertheless, invoking the concept of modularity helps explain two remarkable properties of the catalytic domain, namely (i) its ability to tolerate massive sequence perturbation during evolution and (ii) its ability to evolve diverse modes of allosteric regulation on a common scaffold. Modules within proteins can accommodate mutations without altering the overall protein structure or fold [93]. This is seen in the EPK–ELK-shared module, which is altered in distinct families without apparent change in the structure or fold. Likewise, invoking the concept of modularity provides a plausible explanation for diverse modes of regulation on a common scaffold. For example, new modes of allosteric regulation can be evolved through various combinations of inter-modular linkages. This is illustrated in the case of the CMGC module, which is built upon the EPK module to couple the co-protein-binding insert to the catalytic site, and the AGC module, which couples the regulatory C-terminal tail through modification of the EPK-specific module in the αC-β4 loop. In addition to modularity, conformational flexibility is also a key design feature of the catalytic domain that contributes to its evolvability. Conformational flexibility allows tolerance to mutations and thereby evolution of new functions [97]. This can be appreciated by the occurrence of diverse regulatory motifs in the activation loop that contribute to the unique modes of regulation in individual kinases. Thus, delineating the modules/motifs unique to individual kinases and understanding how they are conformationally coupled to each other will be critical in fully understanding the regulatory diversity and complexity of the kinome.
